# Polygenetic Variants Related to Osteoarthritis Risk and Their Interactions with Energy, Protein, Fat, and Alcohol Intake in Adults in a Large Cohort

**DOI:** 10.3390/diagnostics12020340

**Published:** 2022-01-28

**Authors:** Sunmin Park

**Affiliations:** Food and Nutrition, Obesity/Diabetes Center, Hoseo University, 165 Sechul-Ri, BaeBang-Yup, Asan-Si 31499, Korea; smpark@hoseo.edu; Tel.: +82-41-540-5345; Fax: +82-41-548-0670

**Keywords:** osteoarthritis, immune-related diseases, inflammation, energy, protein, genetic variants

## Abstract

Osteoarthritis (OA) is increasing globally, especially among elderly Asian women, and its increase may be due to the interaction between genetic factors and lifestyle. This study tested the hypothesis that polygenetic variants associated with OA risk interacted with lifestyle in adults over 40 years in the Ansan–Ansung cohort. Genetic variants were chosen through a genome-wide association study with OA participants (case; *n* = 580) and controls without arthritis (*n* = 4850). Genetic variants with interactions were selected by a generalized multifactor dimensionality reduction. The best model’s polygenic risk scores (PRS) were calculated by summing the number of risk alleles in the selected genetic variants. The best five single nucleotide polymorphism (SNP) model included *AIG1*_rs6570550, *COX10*_rs62054459, *DLG2*_rs148643344, *SOX5*_rs73283615, and *PLXNA4*_rs1472529430, while *IL12A*_ rs1491318751 was added to the five-SNP model to produce a six-SNP model. Only *COX10*_rs62054459 in subcutaneous and visceral adipose tissue was associated with COX10 protein expression. The participants, having high-PRS from the five-SNP and six-SNP models, were at a higher OA risk than those with low-PRS by 3.88 and 4.42 times, respectively. The PRS was not associated with metabolic syndrome or with the insulin resistance index (HOMA-IR). Energy, protein, fat, alcohol, and a Western-style diet intake interacted with the PRS to influence OA risk (*p* = 0.005, 0.042, and 0.021, respectively). In the high energy and alcohol intake and low protein, fat, Western-style diet intake, the participants with a high-PRS had a higher incidence of OA than those with low-PRS. In conclusion, the adults with a high-PRS were at a higher OA risk. Particularly, adults with high PRS should have a lower energy intake, higher WSD containing higher protein and fat intake, and moderate alcohol intake to alleviate OA risk. These results can be applied to personalized nutrition plans to decrease OA risk.

## 1. Introduction

The prevalence of degenerative diseases is increasing as the aging population is increasing worldwide. Osteoarthritis (OA) is a degenerative disease that worsens over time and is the most prevalent form of arthritis [1]. OA is a leading cause of disability in older adults. According to the World Health Organization (WHO), the prevalence of OA globally is approximately 9.6% and 18.0% in men and women, respectively. It is increasing rapidly in Asians because the elderly Asian population has increased rapidly from 6.8% in 2008 and is predicted to increase to 16.2% in 2040 [1].

OA is a ‘wear and tear’ disease with chronic pain and inflammation in the joints. The disease is induced by progressive damage to articular cartilage and bone remodeling and is worsened by synovial inflammation, fibrosis of ligaments and tendons, and thickening of the capsules. OA is a multifactorial and complex disease. The unmodifiable risk factors involved in OA are older age, women, and genetics, while the modifiable risk factors are obesity, type 2 diabetes, joint injuries, repeated physical stress on the joints by work and exercise, alcohol consumption, and dietary intake [2,3,4]. Heavy occupational, physically active, and agricultural work increase symptomatic knee and hip OA. Dietary factors influence OA risk by modulating inflammation, oxidative stress, and weight [3,4]. Obesity is a primary risk factor for OA in Western countries, but it is not confirmed in Asians, who are generally not severely obese. Since obesity rates have increased in Asians, obesity will become an essential risk factor in Asia in the future.

Asians generally have a lower risk of OA in most joints, except for the knee joints, than Caucasians [5]. Along with modifiable environmental factors, genetic factors contribute to OA development. Genetic association studies have identified the effects of critical genetic variants on OA pathogenesis. On the other hand, each significant genetic variant has minor impacts, indicating that complex polygenic variants influence OA etiology [2]. Although genetic factors along with age and gender are unmodifiable factors, they interact with lifestyle to influence OA risk. Environmental factors, including lifestyle, interact with genetic factors, and can be applied to personalized nutrition to prevent and manage OA.

Genome-wide linkage scans have identified OA susceptibility genes, such as chromosomes 2, 3, 4, 6, 7, 11, 16, and X. Chromosome 2q13–32 includes the interleukin-1 (*IL-1*) gene cluster, frizzled-related protein 3 (*FRZB*), and cartilage structural protein matrilin-3 (*MATN3*) and is associated with OA risk. Other genes related to OA are alpha1 type IX collagen (*COL9A1*), bone morphogenetic protein 5 (*BMP5*), *IL-4R*, and low-density lipoprotein receptor-related protein 5 (*LRP5*) genes [2]. Similar to the linkage gene study, Gly976Ser and Arg519Cys variations of the Collagen Type II Alpha 1 Chain (*COL2A1*), the primary components of articular cartilage and intervertebral discs, are associated with knee and hip OA risk and reduce the durability of the articular cartilage against mechanical stress [6]. Moreover, the *AA genotype of COL2A1* single-nucleotide polymorphism (SNP) G4006A is positively associated with OA risk, while the TA genotype of T2088C and G4006A haplotype is a positive risk factor for OA in Han Chinese women [7]. On the other hand, few studies on the genetic impact of OA and the interaction between genetic and environmental factors, have been conducted in Koreans. Some potential interactions between OA genetic risk and environmental factors have been reported. Physical activity can modulate gut microbiota composition to promote intestinal mucosal immunity, modify the bile acid profile, and improve the production of short-chain fatty acids by activating a gut-joint axis [8].

This study tested the hypothesis that complex polygenetic variants influenced OA risk and the genetic impact interacted with the lifestyles of Asian adults. This hypothesis was assessed in adults over 40 years in the Ansan–Ansung cohort in Korea. Identifying novel genetic variants and their interaction with lifestyle will eventually provide a better understanding of the OA molecular mechanism, particularly for Asians, and a potential personalized dietary regime for OA prevention and management in the future.

## 2. Methods

### 2.1. Participants

Data collected from 2008–2014 for the Korean Genome and Epidemiology Study (KoGES) were used [9]. Participants were recruited from the rural community of Ansung and the urban community of Ansan city, where they must have resided for at least six months. A total of 5430 participants aged over 40 years (2589 men and 2841 women) were included. All procedures of the KoGES followed the Declaration of Helsinki and were approved by the Institutional Review Boards of the Korean National Institute of Health (1041231-190902-BR-099-01) and Hoseo University (1041231-150811-HR-034-01). Written informed consent was obtained from all participants.

### 2.2. General Characteristics and Biochemical Measurements

The participants were mentally and physically healthy. Age, gender, education, income, physical activity, smoking status, and alcohol consumption were collected during a health interview. Body weight, height, fat, and skeletal muscle mass were measured using Inbody (Inbody 3.0, Biospace, Cheonan, Korea) wearing a light gown [10]. The waist circumference was also measured, 2 cm above the navel, around the waist with a tape measure. The body mass index (BMI) was calculated by dividing the weight (kg) by the square height (m^2^).

Education level was categorized into: less than high school, high school, and college or more. Household income (USD/month) was divided into four groups: very low (<1000), low (1000–2000), intermediate (2000–4000), and high (>4000). Smoking status was categorized into: current smoker, past smoker, and never smoker. A current smoker was defined as having smoked more than 100 cigarettes over the previous six months [11]. Alcohol consumption was assessed by questioning the participants about their drinking behavior during the month before the interview. According to average daily consumption (g/day), the alcohol consumption status was divided into nondrinker, light drinker (1–15), moderate drinker (16–30), and heavy drinker (>30).

Blood pressure was measured on the right arm, in a sitting position, at heart level. Biochemical parameters were determined using plasma and serum from blood drawn after an overnight fast (no foods for more than 12 h) [12]. The lipid profile (total cholesterol, HDL cholesterol, and triglyceride), glucose, aspartate aminotransferase (AST), alanine aminotransferase (ALT), and creatinine concentrations in the circulation were measured using a Hitachi 7600 Automatic Analyzer (Hitachi Ltd., Tokyo, Japan). The white blood cell (WBC) counts and hemoglobin A1c (HbA1c; glycated hemoglobin) concentrations in heparin-treated blood were determined using a Hitachi 7600 Automatic Analyzer (Hitachi, Tokyo, Japan). Serum high-sensitive C-reactive protein (hs-CRP) and insulin concentrations were measured using an ELISA kit (Crystal Chem, Elk Grove Village, IL, USA). The estimated glomerular filtration rate (eGFR) was calculated using the equation as follows: 175 × (serum creatinine concentration)^−1.154^ × (age)^−0.203^. In females, the eGFR was multiplied by 0.742. Homeostatic model assessment for insulin resistance (HOMA-IR) was calculated using the equation as follows: fasting serum insulin (µU/mL) × fasting glucose (mmol/L)/22.5.

### 2.3. Definition of Osteoarthritis, Obesity, and Metabolic Syndrome

An arthritis diagnosis was investigated for a diagnosis of OA or rheumatoid arthritis by a physician. They provided the dates when OA or rheumatoid arthritis was diagnosed by a physician or had the diseases when they enrolled in the study. In the present study, the participants with rheumatoid arthritis were excluded, and the remaining participants were categorized into OA and no osteoarthritis (non-OA) groups. Obesity for Asians is defined as ≥25 kg/m^2^ [13]. Metabolic syndrome was defined according to the 2005 revised National Cholesterol Education Program-Adult Treatment Panel III criteria for Asia, as described previously [14,15].

### 2.4. Assessment of Foods and Nutrient Intake, and Diet Patterns

The semi-quantitative food frequency questionnaire (SQFFQ) designed for Korean diet patterns was assessed for the long-term food intake of the 5430 participants in the Ansan–Ansung studies. The validity and reproducibility of this SQFFQ were evaluated using the four three-day food records for four seasons, similar to the 12-day food records in previous studies in the Korean population [16,17]. The adjusted correlation coefficients between the SQFFQ and 12-day food records ranged from 0.23 and 0.64 in various food intakes, suggesting that the validation and reproducibility of this SQFFQ were acceptable [16,17]. The SQFFQ requested the participant’s average frequency and consumption of 106 food items during the last year. The intake of food frequencies was categorized into never or seldom, once a month, two to three times a month, one to two times a week, three to four times a week, five to six times a week, once a day, twice a day, and three times or more every day. The amount of food intake at once was checked for “more”, “equal”, or “less” based on a regular and defined portion size. The food intake per day in each food category was calculated by multiplying the midpoint of the reported frequency category for each food item by the food amount at once. The daily intake was calculated based on the midpoint of the reported frequency category for each food item. For example, when the frequency of one food item was noted at five-six/week, it was calculated to be 5.5/7 or 0.79 times/day.

The daily nutrient intake was calculated from the food intake from the SQFFQ. The energy and nutrients, such as protein, carbohydrates, fat, fiber, total vitamin A, vitamin C, Na, Ca, and K, were calculated by converting the foods to nutrients using the Can-Pro 2.0 nutrient intake assessment software developed by the Korean Nutrition Society (Seoul, Korea). The daily Estimated Energy Requirement (EER) and recommended nutrient intake were based on the Korean Dietary Reference Intake (DRI) according to age and gender [14].

The 106 food items were divided into 29 food groups used as independent variables during factor analysis to determine the dietary patterns by PCA. The number of factors in the principal component analysis (PCA) were assigned using the eigenvalues > 1.5, and the orthogonal rotation procedure was applied [15]. Three distinct dietary factors were selected, and dietary factor-loading values of ≥0.40 were considered significant contributions to the dietary patterns. The dietary patterns represented Western-style, plant-based, and rice-main diets (Table S1). The Western-style diet was rich in bread, cookies, mushrooms, fish, crabs, meats, processed meats, beverages, and fast food, while the plant-based diet was high in beans, potatoes, kimchi, vegetables, pickles, seaweed, and fruit. The rice-main diet was high in rice only.

### 2.5. Genotyping and Quality Control

The participants’ genotyping and quality-control processes were conducted in the Center for Genome Science, Korea National Institute of Health, as described previously [9]. Briefly, deoxyribonucleic acid (DNA) samples of the participants were isolated from the peripheral blood of the participants and genotyped using the Korean Chip included SNPs involved in the prevalent diseases for Koreans (Affymetrix, Santa Clara, CA, USA). The genotyping accuracy was examined using Bayesian Robust Linear Modeling with the Mahalanobis distance genotyping algorithm [18]. Samples with low genotyping accuracies of <98%, high missing genotype call rates (≥4%), high heterozygosity (>30%), or gender biases were excluded. The Institutional Review Board of the Korean National Institute of Health (KBP-2019-055) and Hoseo University (1041231-150811-HR-034-01) approved the KoGES and the present studies. All participants provided written informed consent, and they provided the quality-controlled genotype data.

### 2.6. Expression Quantitative Trait Locus (eQTL) Analysis

eQTL analysis is a direct approach to identify the candidate gene expression of risk loci. The allele variants are associated with the corresponding gene expression, and eQTL analysis identified the candidate susceptible genes in various diseases. Gene expression of the genetic variants related to OA risk was identified by eQTL analysis in the Genotype-Tissue Expression (GTE) × eQTL calculator (https://gtexportal.org/home/tetyourown (accessed on 15 July 2021)). Because the gene expressions in the articular cartilage were not provided in the GTE × eQTL calculator, they were calculated indirectly in the skeletal muscle and adipose tissues, as they are involved in OA risk.

### 2.7. The Best Model with SNP–SNP Interactions to Influence Osteoarthritis Risk

**Figure 1:**
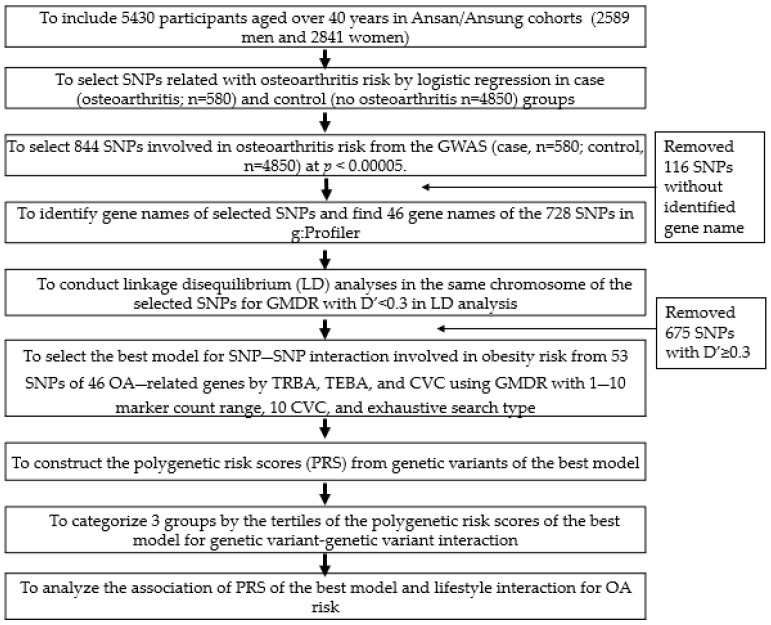
Flow chart for generating polygenetic variants with genetic variant-genetic variant interactions that influence osteoarthritis risk and to explore the interactions between polygenetic risk scores (PRS) and lifestyle.

Figure 1 presents the selection of genetic variants for the best model for genetic variant–genetic variant interactions. The genetic variants explored the association with OA risk by genome-wide association study (GWAS) of the OA and non-OA groups in the Ansan–Ansung cohort. From the GWAS associated with OA risk, 844 genetic variants were selected at *p* < 5 × 10^−5^. The gene names of the 844 SNPs were identified using g:Profiler (https://biit.cs.ut.ee/gprofiler/snpense (accessed on 20 June 2021)), and 53 genes were identified. The 25 obesity-related genes (443 SNPs) in the literature were selected using the Human Genome Epidemiology (HuGE) Navigator (https://phgkb.cdc.gov/PHGKB/hNHome.action (accessed on 29 July 2021)). The corresponding linkage disequilibrium (LD) analyses were carried out on the SNPs of the selected genes in the identical chromosomes using Haploview 4.2 in PLINK. The SNPs with high D’ values (D’ > 0.3) were not included in GMDR because they provided the same information on the genetic impact.

Of the 46 potential genetic variants in the 25 obesity-related genes, ten SNPs exhibiting a SNP–SNP interaction strongly associated with an obesity risk were selected automatically by the generalized multifactor dimensionality reduction (GMDR) analysis. GMDR provided ten potential models among the different combinations of the 46 genetic variants. The best SNP–SNP interaction model was selected in a sign rank test of trained balanced accuracy (TRBA) and testing balanced accuracy (TEBA) with or without adjusting for the covariates using a GMDR program and a *p*-value threshold of 0.05 [19]. The covariates used were age, gender, residence area, education, income, occupation, energy intake, alcohol consumption, regular exercise, and smoking status. Ten-fold cross-validation was also checked for cross-validation consistency (CVC) because the sample size was larger than 1000 [19]; 10 out of 10 in CVC met the perfect cross-validation criteria. Using the best model determined by GMDR analysis, the risk allele of each SNP in the selected best model was counted as 1. For example, when people had AA, AG, and GG in one SNP, and the A allele was the risk allele, the genetic score for the SNP was 2, 1, and 0, respectively. The polygenetic-risk scores (PRS) for the best gene-gene-interaction model were assessed by summing the number of the risk alleles (genetic score) from each selected SNP in the best gene-gene-interaction model [20,21,22]. The PRS in the five and seven SNP models was divided into three categories according to the total number of risk alleles. They were classified as Low-PRS, Middle-PRS, and High-PRS when the number of risk alleles in the PRS was 0–3, 4–5, and ≥6 in the five-SNP model and 0–4, 5–6, and ≥7 in the six-SNP model, respectively.

### 2.8. Statistical Analysis

Statistical analysis was conducted using PLINK version 2.0 (http://pngu.mgh.harvard.edu/~purcell/plink (accessed on 27 May 2021) and SAS version 9.3 (SAS Institute, Cary, NC, USA). Descriptive statistics of the categorical variables (e.g., gender and smoking status) were analyzed using the frequency distributions, and their statistical analysis was evaluated using a Chi-squared test. The descriptive values of the continuous variables were expressed as the means and standard deviations according to the PRS or OA categories. The significance of the differences among the OA or PRS groups was analyzed using a one-way analysis of variance (ANOVA) to adjust the covariates, including age, gender, body mass index, education, income, energy intake (percentage of estimated energy requirement), residence area, daily activity, alcohol intake, and smoking status. Finally, multiple comparisons among the PRS groups were performed using a Tukey’s test.

The associations among the PRSs were obtained using the best model, and the obesity risk was examined using multivariate logistic regression analysis with an adjustment for the covariates. The odds ratios (ORs) and 95% confidence intervals (CI) were calculated as a function of the index reference: Low-PRS. Multivariate logistic regression analysis was conducted using two adjusted models. The covariates of model 1 included gender, age, residence area, education, and income. In contrast, model 2 contained the covariates in model 1 plus smoking status, alcohol consumption, daily energy intake, and regular exercise. The adjusted ORs and 95% CIs were calculated for obesity risk according to PRS.

The participants were categorized into higher and lower intake groups using the above classification criterion. A multivariate interaction model was used to examine the interactions between the PRS and lifestyle and demographic parameters after adjusting for the covariates for model 2. *p*-values < 0.05 were considered significant.

## 3. Results

### 3.1. Characteristics of the Participants According to the Incidence of OA

OA participants were older than those without OA (*p* < 0.001), and participants aged ≥55 years had a 2.5-times higher risk than those aged <55. In the stratification with the age of the participants, OA incidence was elevated with age, and OA risk was higher by 6.4 times in participants aged ≥65 years than those aged <45 years (*p* < 0.001; Table 1). The incidence of OA was much lower in men (19.5%) than women (80.5%) (*p* < 0.001), and women had a 3.2-times higher OA risk than men. The OA participants were diagnosed on average 9.98 years ago, and they still had some pain due to OA (Table 1). The participants with lower education and income had a higher incidence of OA than the others. Education and income were associated with OA risk; higher education and income were inversely associated with OA risk (Table 1).

Height was not significantly different between the OA and control groups. The participants with OA had higher BMI, waist circumference, and fat mass than those without OA (Table 1), but lean body mass did not differ between the OA and non-OA groups. BMI, waist circumference, and fat mass, but not lean body mass, were positively associated with OA risk. Therefore, obesity was associated with OA risk. The serum CRP concentration, an inflammation index, was similar in the OA and non-OA groups (Table 1). The OA group included more participants with metabolic syndrome than the non-OA group, but there was no association between metabolic syndrome and OA risk. The components of metabolic syndrome, including serum glucose, total cholesterol, high-density lipoprotein (HDL), low-density lipoprotein and triglyceride concentrations, systolic blood pressure, and diastolic blood pressure were similar in the OA and non-OA groups. No significant associations of the metabolic syndrome components with OA risk were noted (Table 1). Homeostatic model assessment (HOMA) for insulin resistance (HOMA-IR) and HOMA-β-cell function (HOMA-B) were similar in the OA and non-OA groups. The eGFR was lower in the OA group than the non-OA group, but it was not significantly associated with OA risk (Table 1).

### 3.2. Nutrient Intake and Lifestyles in the OA Participants

The daily energy intake based on the estimated energy intake was similar in the OA and non-OA groups. The macronutrient intake, including carbohydrate, protein, and fat, was similar in the two groups (Table 2). No significant differences in the saturated, monounsaturated, and polyunsaturated fatty acid intake were observed between the OA and non-OA groups. Other nutrient intake, including cholesterol, vitamin C, and fiber intake, did not affect OA risk (Table 2). Unlike the nutrient intake, dietary patterns categorized by principal component analysis (PCA) from the semi-quantitative food frequency questionnaire (SQFFQ) showed a significant difference between the OA and non-OA groups. The dietary patterns were divided into a Western-style diet, plant-based diet, and rice-main diet. Consistent with a high protein diet, the Western-style diet was inversely associated with OA risk (Table 2). However, OA incidence did not differ in a low and high plant-based diet, and rice-main diet groups; and the diet patterns were not significantly associated with OA risk (Table 2). OA incidence was lower in the smoker group than the non-smoker group, while higher in the exercise group than the non-exercise group. However, alcohol consumption, smoking, and regular exercise were not significantly associated with OA risk (Table 2).

### 3.3. Genetic Variants Related to OA Risk and the Best Model with Genetic Variant–Genetic Variant Interaction

After GWAS for OA risk in the Ansan–Ansung cohort and removing some SNPs not meeting the inclusion criteria (Figure 1), only two genetic variants, including androgen induced 1 (*AIG1*)_rs6570550 and cytochrome C oxidase assembly factor heme A (*COX10*)_rs62054459, satisfied the statistical significance of the Bonferroni correction. Fifty-three SNPs containing rs6570550 and rs62054459 were included for determining the best genetic variant–genetic variant interaction model with these two SNPs. Ten SNPs were selected because they showed genetic variant–genetic variant interactions, and 5–10 SNPs in the models met the best model criteria: *p*-value < 0.05 for the sign test of TRBA and TEBA, and CVC = 9 or 10 in GMDR analysis. The characteristics of 10 SNPs are presented in Table 3. The five-SNP model for OA risk included *AIG1*_rs6570550, *COX10*_rs62054459, discs large MAGUK scaffold protein 2 *(DLG2)*_rs148643344, plexin A4 *(PLXNA4)*_rs1472529430, and SRY-box transcription factor 5 *(SOX5)* _rs73283615 (Table S2). The six-SNP model contained the genetic variants in the five-SNP model plus interleukin 12A (*IL12A*)_rs1491318751 (Table S2).

ORs and 95% CI for the PRS of the five- and six-SNP best models of SNP–SNP interaction were 3.89 (2.723–5.548) and 4.42 (3.211–6.09), respectively, in model 2 adjusted for age, gender, BMI, OA duration, the status of smoking and drinking, levels of income and education, job, income, physical activity, energy intake, percentage intake of carbohydrate and fat, and arthritis medication (Table 4). Each SNP selected in the best model was not significantly associated with rheumatoid arthritis, but the PRS of the five-SNP and six-SNP models was significantly associated with the rheumatoid arthritis risk (ORs = 2.041, 95% CI = 1.258–3.311 for the five-SNP and ORs = 1.855, 95% CI = 1.202–2.862) for the six-SNP model. On the other hand, metabolic syndrome and HOMA-IR, which have potentially related etiologies, were not significantly associated with the PRS of the five-SNP and six-SNP models (Table 4).

### 3.4. eQTL Analysis in Skeletal Muscle and Adipose Tissue

**Figure 2:**
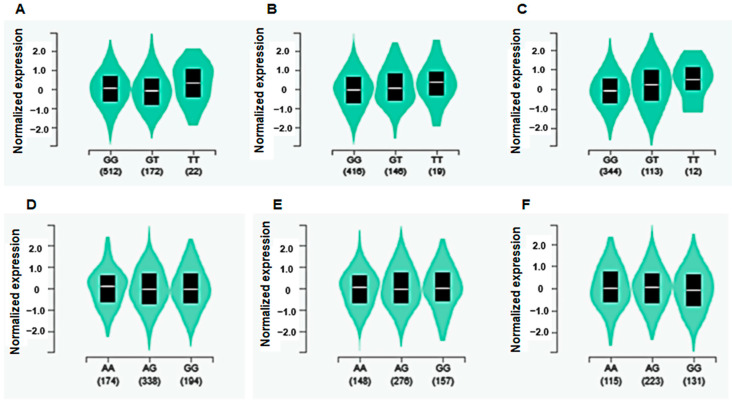
Gene expression of *COX10*_rs62054459 and *AIG1*_rs6570550 in the skeletal muscle, subcutaneous and visceral adipose tissue by eQTL analysis. Gene expression of the genetic variants to influence OA risk was identified by eQTL analysis in the Genotype-Tissue Expression (GTE) × eQTL calculator. (**A**). *COX10*_rs62054459 in the skeletal muscle (*p* = 0.47); (**B**). *COX10*_rs62054459 in subcutaneous adipose tissue (*p* = 0.015); (**C**). *COX10*_rs62054459 in visceral adipose tissue (*p* = 0.014); (**D**). *AIG1*_rs6570550 in skeletal muscle (*p* = 1.0); (**E**). *AIG1*_ rs6570550 in subcutaneous adipose tissue (*p* = 0.41); (**F**). *AIG1*_rs6570550 in visceral adipose tissue (*p* = 0.062).

Among the 10 genetic variants related to OA risk, *COX10*_ rs62054459 had a significant eQTL association. The minor allele of *COX10*_rs62054459 had a higher expression of *COX10* in subcutaneous and visceral adipose tissue but not the skeletal muscle, than its major allele (Figure 2). *AIG1* showed a tendency to an eQTL association, but it was not significantly associated (Figure 2).

### 3.5. Interaction of PRS and Nutrient Intake in OA Risk

**Figure 3:**
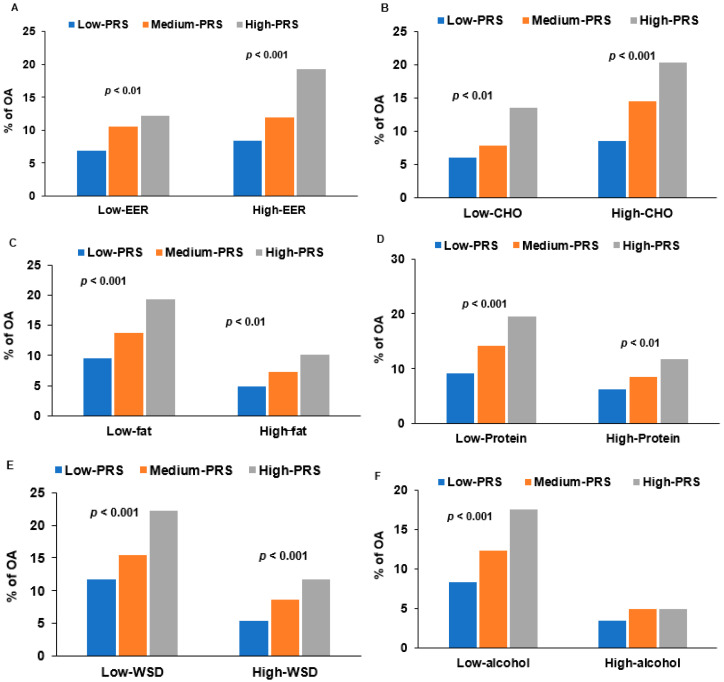
Interaction of nutrient intake with low, medium, or high polygenetic risk scores (PRS) from the 5-SNP model to influence osteoarthritis (OA) risk. (**A**). OA incidence (%) of the participants categorized by daily energy intake (cutoff value: estimated energy requirement (EER) according to age and genders). (**B**). OA incidence (%) of the participants categorized by carbohydrate (CHO) intake (cutoff value: 70 energy percent). (**C**). OA incidence (%) of the participants categorized by fat intake (cutoff value: 15 energy percent). (**D**). OA incidence (%) of the participants categorized by protein intake (cutoff value: 13 energy percent). (**E**). OA incidence (%) of the participants categorized by Western-style diet intake (cutoff value: 70th percentile). (**F**). OA incidence (%) of the participants by alcohol intake (cutoff value: 20 g alcohol/day). Covariates included age, gender, body mass index, OA duration, the status of smoking and drinking, levels of income and education, job, income, physical activity, energy intake, percent intake for carbohydrate and fat, and arthritis medication.

Although the participants were diagnosed with OA approximately 9.98 ± 0.39 years before enrolling in the Ansan–Ansung cohort study, they had OA symptoms when they enrolled in the Ansan–Ansung cohort study. The dietary intake involved OA symptoms by interacting with the PRS. The PRS from the five-SNP and six-SNP models showed a similar interaction, and the interaction results of the PRS were provided from the five-SNP model. The energy intake interacted with the PRS to influence OA risk (*p* = 0.0048): in high energy intake, OA risk was much higher in the high-PRS group than in the low-PRS group (Table 5 and Figure 3A). Among energy intake, carbohydrate intake did not interact with PRS to influence OA risk (Table 5), and OA incidence increased with PRS in both high- and low-CHO groups (Figure 3B). The protein and fat intakes showed an interaction with the PRS to affect OA risk; in the low protein (*p* = 0.0367) and fat intake (*p* = 0.0420), the participants with a high-PRS had a much higher OA incidence than those with a low-PRS (Table 5 and Figure 3C,D). As with the high protein diet, WSD had an interaction with PRS (*p* = 0.0304; Table 5), and the increase of OA incidence with PRS was lower in a high WSD than in a low WSD (Figure 3E). However, a plant-based and rice-main diet did not interact with the PRS to influence OA risk (Table 5). Alcohol intake also interacted with the PRS for OA risk: OA incidence was much higher in the participants with high-PRS than low-PRS in the participants with a low alcohol intake, but not those with a high alcohol intake (Table 5 and Figure 3F). Regular exercise and smoking status did not interact with the PRS, although OA incidence was positively associated with PRS in both low- and high-exercise groups and smoking status (Table 5).

## 4. Discussion

OA is a complex polygenetic disease involving genetic and environmental factors that influence OA risk. The incidence of OA is increasing worldwide as the elderly population increases; its increase is significant in older Asian women. Arthritis is a broad name for joint pain and inflammation, but the causes and etiologies are different. OA develops with wear and tear in the articular cartilage of the joints by repeated stress with aging. The symptoms are similar in all cases of arthritis, but the etiology is different. The present study determined the genetic factors for OA risk in the Ansan–Ansung cohort because this cohort included the incidence of arthritis divided into OA and rheumatoid arthritis. On the other hand, the city hospital-based cohort, the larger cohort in KoGES, included arthritis incidence, but it was not separated into OA and rheumatoid arthritis. The present study is novel because it explores the polygenetic impact on OA and its interaction with nutrient intake in the Ansan–Ansung cohort.

OA develops slowly with age and is accompanied by pain, stiffness, loss of flexibility, grating sensation, bone spurs, and swelling. The disease is involved in the gradual degradation of the extracellular matrix in cartilage and bone. Cartilage is composed mainly of collagen type 2 with aggrecan and proteoglycans. The environmental risk factors for OA have been well established, including older age, women, obesity, repeated stress on the joints, joint injuries, bone deformities. Genetic factors affecting OA development have been reported, but they remain unclear. A collagen type 2 gene mutation is involved directly in OA incidence in a case report [23]. Cartilage is maintained by its synthesis and degradation, and genetic factors are related. Its degradation is involved in extracellular matrix (ECM) degradation enzymes, such as matrix metalloproteinase (MMP)-1, MMP-3, and MMP-13, in chondrocytes and ECM synthesis by the *SOX9* expression [24]. However, their genetic variants were not selected for OA risk in the present study.

Obesity is positively associated with OA risk. It is not just due to the heavy load on the knee due to high BMI, but due to increased inflammation by increased adipose tissue [2,3]. The present study demonstrated that fat mass was positively associated with OA risk but not lean body mass. OA is involved in inflammation stimuli in synovium inflammation and cartilage degradation through the catabolic activities of chondrocytes [25]. Inflammatory cytokines, including IL-1, IL-4, IL-6, IL-17, IL-18, and tumor necrosis factor (TNF)-β, stimulate matrix degradation [26]. *IL-12* regulates the balance between Th1 and Th2 cells and enhances cytotoxic T cell-mediated lysis and natural killer (NK) cell activity. The *IL-12* mRNA and protein levels are higher in rheumatoid arthritis and OA joints than in normal adults [25,27]. In the present study, *IL12A*_rs1491318751 was significantly and positively associated with OA. It is also related to collagen synthesis in the cartilage, while SOX5 is involved in enhanced chondrogenic differentiation and chondrogenesis, and SOX5 suppression, with miR-194 up-regulation, decreases chondrogenesis [28]. In the present study, a person with the minor *SOX5*_rs73283615 allele was positively associated with OA risk. Therefore, the genetic impact might be related to inflammation and chondrogenic differentiation in OA in Korean adults. Furthermore, the neuronal network contributes to OA-induced pain to exacerbate OA symptoms. In the present study, genetic variants of *SOX5, PLXNA4*, and *DLG2* involved in neuronal networks were involved in OA risk. Therefore, OA risk is associated with synovium inflammation, cartilage degradation, inflammation, and the neuronal network.

*COX10*_rs62054459 and *AIG1*_rs6570550 met the statistical significance of the Bonferroni correction (*p* < 5 × 10^−8^), and were included in the best model for OA genetic risk in the present study. People with the *AIG1*_rs6570550 minor allele were positively associated with OA risk, while those with *COX10_* rs62054459 minor alleles were inversely associated with it. *COX10* is the terminal component in the mitochondria respiratory chain reaction for the electron transfer from reduced cytochrome C to oxygen. *COX10* knockout mice began to develop progressive myopathy at three months of age, and the myopathy was worsened by aging, particularly in female mice [29]. On the other hand, no study has examined the effects of a *COX10* mutation on OA risk. *COX10* missense mutation is associated with mitochondrial dysfunction. In the present study, the participants with the minor *COX10*_rs62054459 allele were inversely associated with OA risk. *COX10* gene expression in the articular cartilage were not provided according to *COX10*_rs62054459. However, because OA is related to skeletal muscle and adipose tissues, their *COX10* expression levels were determined. Interestingly, *COX10* expression in people with the rs62054459 minor allele had higher expression in the skeletal muscle, subcutaneous adipose tissue, and visceral adipose tissue in eQTL analysis. This suggests that people with the rs62054459 minor allele might have better mitochondrial function. Therefore, people with the *COX10* rs62054459 minor allele might have a protective effect in OA by increasing *COX10* expression.

The association of AIGI with OA has not been studied. AIGI acts as a hydrolase of fatty acid esters of hydroxy fatty acids located in the integral membrane, but not other lipids [30]. It is expressed at higher levels in the heart, ovary, testes, liver, kidney, small intestine, and colon and lower levels in the spleen, prostate, brain, skeletal muscle, and pancreas. *AIG1* expression in articular cartilage has not been reported. The present study showed that *AIG1* expression was lower in those with the minor allele *AIG1*_rs6570550, than those with the major allele in eQTL analysis. Furthermore, the *AIGI* genetic variant effect may be involved in gender differences in OA incidence, as women have a much higher incidence of OA than men. Decreased *AIG1* expression, due to having the minor allele *AIG1*_rs6570550, might be associated with OA risk.

OA risk is affected by lifestyle, but few regimes efficiently prevent or reduce the it [31]. Fish oil, protein, dietary fiber, and polyphenols, including curcumin, are recommended to improve OA symptoms [32,33,34,35]. On the other hand, their efficacy is varied and controversial [36]. OA risk is closely related to obesity, and weight loss is recommended for reducing OA symptoms. Intermittent fasting is one weight-loss method and OA therapy [33]. The present study showed that PRS interacted with dietary intake, including energy, protein, fat, and alcohol, to influence OA risk, even though dietary intake did not affect OA risk. In the present study, adults with high-PRS demonstrated a higher OA risk with high-energy intake and low protein and fat intake. On the other hand, dietary patterns did not significantly affect OA risk. Therefore, adults aged >40 years might need a lower energy intake with nutritious foods, including high protein, and maintain body weight within a normal range to prevent or alleviate OA symptoms.

Polygenetic variants including *COX10* and *AIG1* were associated with OA risk, and the impact of the two genetic variants on OA was related to their gene expression. As COX10 and AIG1 are involved in energy and sex hormone metabolism, respectively, the present study indirectly demonstrated the genetic impact of the energy metabolism and gender relationship in OA risk. The present study demonstrated that BMI, waist circumference, and fat mass, but not lean body mass, were positively associated with OA risk, and PRS and energy intake had an interaction showing that low energy intake decreased OA risk primarily in participants with high-PRS. Genetic variants of *COX10* and *AIG1* were indirectly involved in OA risk through modulating energy metabolism. Additionally, the minor-alleles of *COX-10* modulated the COX-10 protein expression to influence OA risk. Therefore, genetic variants involved in energy, inflammation, and neuronal networks are associated with OA risk.

The present study is the first to determine genetic variants to show that PRS–PRS and PRS–lifestyle interactions influence OA risk. Nevertheless, the present study had some limitations. First, this was a case-control study to collect data cross-sectionally. Second, OA incidence was reported by a questionnaire of an OA diagnosis by a physician, even though the rheumatoid arthritis incidence was asked separately. Third, gene expression according to genetic variant alleles was not determined in the articular cartilage. Finally, lifestyle and nutrient intake were self-reported with questionnaires and may have included some memory biases.

In conclusion, PRS generated from the five-SNP best model with SNP–SNP interaction for OA risk included *AIG1*_rs6570550, *COX10*_rs62054459, *DLG2*_rs148643344, *PLXNA4*_ rs1472529430, and *SOX5*_rs73283615. After adjusting for the covariates related to OA risk, the high-PRS participants were 3.89 times more positively associated with OA risk. The PRS did not have an association with metabolic syndrome and insulin resistance. Energy, protein, and fat intake interacted with PRS to influence OA risk. In the high energy, low protein, and low-fat intake groups, the PRS impact on OA incidence was much higher than in the low intake group. In taking together nutrient intake, the high-WSD lowered OA incidence more than the low-WSD in contrast to metabolic syndrome risk. Therefore, adults with a high-PRS should consume less energy than EER and have a high protein and moderate fat intake to prevent or alleviate OA symptoms. The PRS data can be applied to personalized nutrition for preventing OA.

## Figures and Tables

**Table 1:** Characteristics of the participants with osteoarthritis.

	Control (*n* = 4850)	Osteoarthritis (*n* = 580)	Adjusted ORs and 95% CI
Age (<55 yr)	51.1 ± 0.10 ^1^	54.7 ± 0.32 ***	2.501 (1.989~3.144)
<45	1428 (96.8)	48 (3.25) ***	1
45–54	1826 (92.0)	158 (7.96)	2.388 (1.663~3.427)
55–64	1176 (82.4)	251 (17.6)	4.344 (2.983~6.326)
≥65	420 (8.66)	123 (22.6)	6.415 (4.203~9.790)
Sex (N, %; males)	2476 (51.1)	113 (19.5) ***	3.198 (2.257~4.531)
Osteoarthritis duration (yrs)	0 ± 0	9.98 ± 0.39	
Height (cm)	160.3 ± 0.08	160.4 ± 0.24	1.098 (0.805~1.498)
BMI (<25 kg/m^2^)	24.5 ± 0.05	25.4 ± 0.14 ***	1.488 (1.180~1.876)
Waist circumferences (M: <90 cm; F: <85 cm)	82.2 ± 0.12	84.6 ± 0.37 ***	1.372 (1.106~1.703)
Lean body mass (M: <35%; F: <28%)	31.1 ± 0.05	31.5 ± 0.12	1.148 (0.926~1.424)
Fat mass (M: <25%; F: <30%)	26.5 ± 0.08	27.7 ± 0.26 ***	1.246 (1.005~1.545)
Education (N, %)			
≤Middle school	2421 (84.3)	452 (15.7) ***	1
High school	1687 (94.4)	100 (5.60)	0.711 (0.531~0.951)
≥College	723 (96.8)	24 (3.21)	0.605 (0.376~0.974)
Income (N, %)			
≤$2000	1421(81.8)	316 (18.2) ***	1
$2000–4000	2398 (92.2)	203 (7.80)	0.777 (0.621~1.001)
>$4000	971 (95.3)	48 (4.71)	0.690 (0.469~1.015)
MetS (N, %)	908 (16.7)	173 (29.8) ***	1.051 (0.814~1.356)
Serum glucose (<126 mg/dL)	87.5 ± 0.30	86.7 ± 0.93	0.864 (0.659~1.135)
Serum insulin (<9.5 IU/L)	7.48 ± 0.06	7.63 ± 0.19	0.936 (0.760~1.154)
HbA1c (<6.5%)	5.78 ± 0.01	5.72 ± 0.04	0.967 (0.717~1.305)
HOMA-IR (<1.95)	1.63 ± 0.02	1.65 ± 0.04	0.969 (0.772~1.216)
HOMA-B (<160)	149.4 ± 2.09	150.9 ± 6.50	1.109 (0.905~1.361)
Serum total cholesterol (<230 mg/dL)	192.6 ± 0.51	194.4 ± 1.57	0.995 (0.762~1.300)
Serum HDL (M: <40, F: <50 mg/dL)	44.7 ± 0.14	44.6 ± 0.43	0.984 (0.791~1.225)
Serum LDL (<130 mg/dL)	115.6 ± 0.48	118.0 ± 1.49	1.049 (0.760~1.447)
Serum Triglyceride (<150 mg/dL)	161.7 ± 1.51	159.1 ± 4.66	1.008 (0.822~1.235)
Serum CRP (<0.5 mg/dL)	0.22 ± 0.01	0.21 ± 0.02	0.841 (0.451~1.571)
SBP (<130 mmHg)	116.6 ± 0.24	117.0 ± 0.73	0.908 (0.723~1.140)
DBP (<90 mmHg)	75.0 ± 0.16	75.1 ± 0.48	1.016 (0.760~1.358)
eGFR (<70 mL/min)	85.4 ± 0.23	83.8 ± 0.71 *	1.021 (0.793~1.314)
Serum AST (<40 U/L)	29.2 ± 0.26	28.7 ± 0.81	0.880 (0.586~1.322)
Serum ALT(<35 U/L)	28.3 ± 0.45	27.8 ± 1.38	1.051 (0.791~1.396)

^1^ Adjusted means and 95% confidence intervals after adjusting for covariates included age, gender, education, income, energy intake (percentage of estimated energy requirement), residence area, daily activity, alcohol consumption, and smoking status. * Significantly different from the control group at *p* < 0.05 and *** at *p* < 0.001.

**Table 2:** Lifestyle including nutrient intake and association with obesity in the participants according to genders and obese status.

	Control (*n* = 4850)	Osteoarthritis (*n* = 580)	Adjusted ORs and 95% CI
Energy (<EER%) ^1^	102.7 ± 0.53 ^2^	105.4 ± 1.65	1.175 (0.966~1.428) ^3^
Carbohydrates (<70 En%)	70.8 ± 0.09	70.8 ± 0.29	1.055 (0.836~1.332)
Fiber (<20 g/d)	21.3 ± 0.18	21.7 ± 0.56	0.966 (0.771~1.211)
Protein (<13 En%)	13.5 ± 0.03	13.6 ± 0.10	0.957 (0.775~1.183)
Fat (<15 En%)	14.6 ± 0.07	14.5 ± 0.22	0.975 (0.772~1.230)
Saturated fat (<5.7 En%)	4.2 ± 0.4	4.3 ± 1.1	1.045 (0.798~1.368)
Monounsaturated fat (<7.0 En%)	5.4 ± 0.4	5.5 ± 1.2	0.979 (0.737~1.299)
Polyunsaturated fat (<3.5 En%)	2.6 ± 0.2	2.6 ± 0.5	0.880 (0.640~1.210)
Cholesterol (<250 mg/d)	177 ± 1.57	179 ± 4.85	0.936 (0.705~1.243)
Vitamin C (<100 mg/d)	128 ± 1.13	126 ± 3.50	0.932 (0.701~1.239)
Plant-based diet (<70th percentile)	1588 (32.7) ^4^	211 (36.4)	1.180 (0.944~1.476)
Western-style diet (<70th percentile)	1691 (34.9)	113 (19.5) ***	0.726 (0.550~0.957)
Rice-main diet (<70th percentile)	1623 (33.4)	173 (29.8)	1.081 (0.866~1.349)
Flavonoids (<70th percentile)	64.2 ± 0.82	61.8 ± 2.56	0.965 (0.758~1.228)
Alcohol drinking (<20 g/d)	9.85 ± 0.29	10.2 ± 0.90	1.061 (0.702~1.602)
Smoking (current smokers)	1220 (25.6)	58 (10.2) ***	0.802 (0.501~1.285)
Regular exercise (<150 min/week)	1379 (29.2)	224 (40.5) ***	1.212 (0.955~1.538)

^1^ The cutoff points for logistic regression. ^2^ The values represent adjusted means ± standard errors. ^3^ Adjusted odds ratio (ORs) and 95% confidence intervals after adjusting for covariates included age, gender, education, income, energy intake (percentage of estimated energy requirement), residence area, daily activity, alcohol consumption, and smoking status. ^4^ The number of the subjects (percentage of each group). *** Significantly different from the control group at *** *p* < 0.001.

**Table 3:** The characteristics of the ten genetic variants of genes related to osteoarthritis in adults using the generalized multifactor dimensionality reduction analysis.

CHR ^1^	SNP ^2^	Location	Mi ^3^	OR ^4^	L95 ^5^	U95 ^6^	*p*-Value for OR ^7^	Genes	Feature	MAF ^8^	HWE ^9^
3	rs149045369	129206303	T	0.324	0.1925	0.5449	2.16 × 10^−5^	*IFT122*	transcript	0.0526	0.786
3	rs1491318751	159765533	G	1.639	1.3	2.067	2.94 × 10^−5^	*IL12A*	intron	0.0974	1
6	rs6913416	157454046	C	2.559	1.659	3.947	2.14 × 10^−5^	*ARID1B*	intron	0.0194	1
6	rs6570550	143480314	A	1.572	1.338	1.847	3.73 × 10^−8^	*AIG1*	intron	0.3139	0.117
7	rs1472529430	132018047	T	0.602	0.4785	0.7564	1.37 × 10^−5^	*PLXNA4*	intron	0.1802	1
11	rs148643344	85026573	G	1.765	1.379	2.258	6.29 × 10^−6^	*DLG2*	intron	0.0771	0.128
12	rs73283618	24112286	C	1.419	1.214	1.658	1.05 × 10^−5^	*SOX5*	intron	0.3767	0.752
17	rs62054459	13672047	T	0.567	0.4626	0.6939	3.96 × 10^−8^	*COX10*	intron	0.232	0.733
17	rs138377463	43069398	A	1.927	1.401	2.65	5.43 × 10^−5^	*NMT1*	intron	0.0434	0.518
20	rs141079635	41491626	C	2.077	1.482	2.912	2.18 × 10^−5^	*PTPRT*	intron	0.0382	0.357

^1^ Chromosome; ^2^ Single nucleotide polymorphism; ^3^ Minor allele; ^4^ Odds ratio; ^5^ Lower and ^6^ upper ends of 95% confidence interval; ^7^ *p*-value for OR after adjusting for age, gender, residence area, survey year, body mass index, daily energy intake, levels of education and income; ^8^ Minor allele frequency; ^9^ Hardy–Weinberg equilibrium.

**Table 4:** Adjusted odds ratios for osteoarthritis according to the polygenetic risk scores of the best model (PRS) for gene–gene interaction after covariate adjustments.

	Model 1			Model 2	
For 5-SNP Model	Low-PRS (*n* = 2373) ^1^	Medium-PRS (*n* = 1583)	High-PRS (*n* = 1474)	Medium-PRS (*n* = 1583)	High-PRS (*n* = 1474)
Osteoarthritis	1	2.332 ^2^ (1.853~2.935) ^2^	3.708 (2.617~5.254)	2.381 (1.876~3.021)	3.887 (2.723~5.548)
Rheumatoid arthritis	1	1.309 (0.978~1.752)	1.807 (1.128~2.894)	1.316 (0.961~1.801)	2.041 (1.258~3.311)
Metabolic syndrome	1	0.908 (0.773~1.067)	0.910 (0.679~1.218)	0.886 (0.744~1.055)	0.855 (0.624~1.169)
HOMA-IR	1	1.063 (0.945~1.196)	1.028 (0.829~1.274)	1.080 (0.952~1.225)	1.030 (0.821~1.293)
For 6-SNP model	Low-PRS (*n* = 2100) ^3^	Medium~PRS (*n* = 2658)	High~PRS (*n* = 672)	Medium~PRS (*n* = 2658)	High~PRS (*n* = 672)
Osteoarthritis	1	2.062 (1.619~2.626)	4.165 (3.051~5.687)	2.087 (1.624~2.681)	4.422 (3.211~6.09)
Rheumatoid arthritis	1	1.173 (0.867~1.585)	1.735 (1.146~2.626)	1.181 (0.857~1.627)	1.855 (1.202~2.862)
Metabolic syndrome	1	0.912 (0.772~1.078)	0.841 (0.656~1.078)	0.879 (0.732~1.056)	0.802 (0.611~1.053)
HOMA-IR	1	1.027 (0.910~1.160)	0.974 (0.810~1.170)	1.052 (0.925~1.198)	0.977 (0.803~1.190)

^1^ Number of participants in each PRS category of the 5-SNP model: Low-PRS (<4), Medium-PRS (4–6), High-PRS (>6) for 5-SNP model. ^2^ Values were expressed as odds ratio and 95% confidence intervals after adjusting for covariates including age, gender, education, income, and residence areas for model 1, parameters of model 1 plus energy intake (percentage of estimated energy requirement), physical activity, alcohol intake, and smoking status for model 2. ^3^ Number of participants in each PRS category of the 6-SNP model: Low-PRS (<5), Medium-PRS (5–7), High-PRS (>7) for 6-SNP model.

**Table 5:** Adjusted odds ratios for osteoarthritis risk by polygenetic risk scores of the 5-SNP best model (PRS) for gene–gene interaction after covariate adjustments according to lifestyle patterns.

	Low-PRS (*n* = 2373) ^1^	Medium-PRS (*n* = 1583)	High-PRS (*n* = 1474)	PRS-Nutrient Interaction *p*-Value ^3^
Low energy	1	1.797 (1.290~2.503) ^2^	3.010 (1.937~4.678)	0.0048
High energy ^4^		1.934 (1.400~2.673)	5.137 (3.396 ~7.770)	
Low carbohydrate	1	1.631 (1.091~2.439)	3.567 (2.200~5.785)	0.0864
High carbohydrate ^5^		1.905 (1.450~2.502)	3.878 (2.677~5.616)	
Low protein	1	1.979 (1.462~2.678)	4.201 (2.794~6.317)	0.0367
High protein ^6^		1.619 (1.154~2.272)	3.385 (2.217~5.168)	
Low fat	1	1.751 (1.343~2.283)	3.838 (2.682~5.494)	0.0420
Moderate fat ^7^		2.019 (1.315~3.100)	3.641 (2.168~6.117)	
Low alcohol	1	1.865 (1.474~2.360)	4.011 (2.948~5.459)	0.0207
High alcohol ^8^		1.333 (0.603~2.948)	2.403 (0.907~6.366)	
Low WSD	1	1.742 (1.249~2.430)	4.127 (2.784~6.116)	0.0304
High WSD ^9^		1.949 (1.413~2.690)	3.776 (2.361~6.039)	
Low PBD	1	2.244 (1.478~3.409)	4.103 (2.362~7.128)	0.5343
High PBD ^10^		1.729 (1.309~2.282)	3.855 (2.698~5.508)	
Low RMD	1	1.555 (1.070~2.261)	3.817 (2.350~6.198)	0.0591
High RMD ^11^		2.079 (1.548~2.793)	4.015 (2.741~5.881)	
Low exercise	1	2.163 (1.601~2.922)	4.633 (3.167~6.778)	0.1367
High exercise ^12^		1.484 (1.051~2.096)	2.904 (1.820~4.635)	
Non-smoker	1	1.882 (1.478~2.395)	4.583 (3.349~6.270)	0.1207
Smoker + former smoker		1.721 (1.363~2.174)	3.719 (2.735~5.056)	

^1^ PRS was divided into three categories (0–3, 4–6, and >6) by three groups as the low, medium, and high groups of the 5-SNP best model of GMDR. Low-PRS was the reference. ^2^ Values were expressed as odds ratio and 95% confidence intervals. ^3^ Multivariate regression models include the main effects, interaction terms of gene and main effects (energy and nutrient intake), and potential confounders including sex, age, BMI, the status of smoking and drinking, levels of income and education, job, physical activity, hypertension, energy, milk, percent intake of carbohydrate and fat, and medication for arthritis and dermatitis. The cutoff points of the parameters were defined as: ^4^ <estimated energy intake, ^5^ <70% carbohydrate, ^6^ <13% protein, ^7^ <15% fat, ^8^ <20 g/day alcohol, ^9–11^ Western-style diet (WSD), plant-based diet (PBD), and rice-main diet (RMD), respectively, and ^12^ 150 min moderate exercise per week.

## Data Availability

The data that support the findings of this study are available from the corresponding author upon reasonable request.

## Supplementary Materials

The following supporting information can be downloaded at: https://www.mdpi.com/article/10.3390/diagnostics12020340/s1. Table S1: Factor loadings of food groups in dietary patterns identified using principal component analysis. Table S2: The characteristics of the ten genetic variants of genes related to osteoarthritis (OA) in adults.
