# Supplementary material for: Polygenetic Variants Related to Osteoarthritis Risk and Their Interactions with Energy, Protein, Fat, and Alcohol Intake in Adults in a Large Cohort
Source: Diagnostics (Basel). 2022 Jan 28;12(2):340. doi: 10.3390/diagnostics12020340 (PMC8871305; doi:10.3390/diagnostics12020340)
Supplement: Supplementary file 1 [file diagnostics-12-00340-s001.zip › diagnostics-1546609-supplementary.pdf]

Supplemental Table S1. Factor loadings of food groups in dietary patterns identified using principle component analysis

	Western-style diet		Plant-based diet		Rice-main diet	
Rice	0		8		90	*
Grains	-7		10		-89	*
Noodles	24		19		10	
Breads	42	*	5		-4	
Cookies	53	*	1		6	
Beans	19		40		-24	
Potatoes	-13		75	*	6	
Kimchi	-23		74	*	10	
Eggs	28		23		-1	
Fast foods	38		-8		-6	
Green vegetables	33		54	*	-9	
White vegetables	27		57	*	-3	
Mushroom	44	*	23		-13	
Fatty fish	51	*	21		-5	
White fish	44	*	29		-7	
crab	51	*	25		-5	
Processed meats	57	*	11		0	
Red meats	59	*	24		17	
Chicken	52	*	15		11	
Seaweed	39		40	*	-16	
Milk and dairy products	39		7		-14	
Beverages	46	*	12		0	
Coffee	25		-4		14	
Tea	22		6		-20	
Fruits	22		46	*	-2	
Pickles	14		42	*	-3	
Nuts	26		9		-10	
**Variance Explained by Each Factor**	4.747		1.900		1.801	

Printed values are multiplied by 100 and rounded to the nearest integer. Values greater than 0.4 are flagged

Supplemental Table S2. The characteristics of the ten genetic variants of genes related to osteoarthritis (OA) in adults

**GMDR (10 SNP)**	Adjusted for sex, age, area, education, BMI, OA duration				Adjusted for sex, age, area, education, BMI, smoking, alcohol, exercise, DM, OA duration			
Model	TRBA	TEBA	P value	CVC	TRBA	TEBA	P value	CVC
*COX10*_rs62054459	0.5512	0.5523	10 (0.001)	10/10	0.5513	0.5523	10 (0.001)	10/10
*AIG1*_rs6570550 model 1	0.5675	0.5595	9 (0.017)	8/10	0.5675	0.5594	9 (0.017)	8/10
*DLG2*_rs148643344 model 2	0.5845	0.5624	10 (0.001)	6/10	0.5843	0.5626	10 (0.001)	5/10
*PLXNA4*_rs1472529430 model 3	0.6015	0.5580	9 (0.017)	6/10	0.6014	0.5552	9 (0.017)	7/10
*SOX5*_rs73283615 model 4	0.6229	0.5590	10 (0.001)	10/10	0.6228	0.5602	10 (0.001)	10/10
*IL12A*_rs1491318751 model 5	0.6458	0.5545	10 (0.001)	10/10	0.6456	0.5597	10 (0.001)	10/10
*PTPRT*_rs141079635 model 6	0.6686	0.5624	10 (0.001)	9/10	0.6684	0.5597	10 (0.001)	9/10
*NMT1*_rs138377463 model 7	0.6884	0.5650	10 (0.001)	9/10	0.6883	0.5657	10 (0.001)	9/10
*IFT122*_rs149045369 model 8	0.7049	0.5679	10 (0.001)	10/10	0.7048	0.5638	10 (0.001)	10/10
ARID1B_rs6913416 model 9	0.7111	0.5526	10 (0.001)	10/10	0.7110	0.5636	10 (0.001)	10/10

TRBA, trained balanced accuracy; TEBA, test balance accuracy; CVC, cross-validation consistency; P-value for the significance of GMDR model by sign test with and without adjusting for covariates designated in the table. *COX10,* cytochrome C oxidase assembly factor heme A:farnesyltransferase; *AIG1,* androgen induced 1; DLG2, discs large MAGUK scaffold protein 2; *PLXNA4,* plexin A4; *SOX5,* SRY-box transcription factor 5; *IL12A,* interleukin 12A; *PTPRT,* protein tyrosine phosphatase receptor type T; *NMT1,* N-myristoyltransferase 1; *IFT122,* intraflagellar transport 122; *ARID1B,* AT-rich Interaction domain 1B.
